# RNA origami: design, simulation and application

**DOI:** 10.1080/15476286.2023.2237719

**Published:** 2023-07-27

**Authors:** Erik Poppleton, Niklas Urbanek, Taniya Chakraborty, Alessandra Griffo, Luca Monari, Kerstin Göpfrich

**Affiliations:** a Biophysical Engineering Group, Center for Molecular Biology of Heidelberg University (ZMBH), Heidelberg University, Heidelberg, Germany; b Biophysical Engineering Group, Max Planck Institute for Medical Research, Heidelberg, Germany; c Molecular Biomechanics, Heidelberg Institute for Theoretical Studies (HITS), Heidelberg, Germany; d Institut de Science Et D’ingénierie Supramoléculaires (ISIS), Université de Strasbourg, Strasbourg, France

**Keywords:** RNA origami, RNA nanotechnology, DNA origami, co-transcriptional folding, RNA nanostructures, genetic encoding, computational design, molecular simulation

## Abstract

Design strategies for DNA and RNA nanostructures have developed along parallel lines for the past 30 years, from small structural motifs derived from biology to large ‘origami’ structures with thousands to tens of thousands of bases. With the recent publication of numerous RNA origami structures and improved design methods-even permitting co-transcriptional folding of kilobase-sized structures – the RNA nanotechnolgy field is at an inflection point. Here, we review the key achievements which inspired and enabled RNA origami design and draw comparisons with the development and applications of DNA origami structures. We further present the available computational tools for the design and the simulation, which will be key to the growth of the RNA origami community. Finally, we portray the transition from RNA origami structure to function. Several functional RNA origami structures exist already, their expression in cells has been demonstrated and first applications in cell biology have already been realized. Overall, we foresee that the fast-paced RNA origami field will provide new molecular hardware for biophysics, synthetic biology and biomedicine, complementing the DNA origami toolbox.

## Growing big: A brief history of RNA origami

### The beginning: tectoRNA complexes

With the identification of RNA structures with enzymatic activity in the early 1980s [1,2], researchers realized that RNA was not just a transient information carrier, but a functional partner in the biochemistry of the cell. This led to many designs of synthetic ribozymes based on the features of the *Tetrahymena* group I intron [3], or the hammerhead ribozyme [4]. These structures, however, were limited to modifications of the naturally occurring structures and the goal of developing *de novo* RNA architectures remained. Inspired by the 3D organization of protein domains and early work in DNA nanotechnology [5; 6] (which in at least one case was mapped to RNA [7,8]), the first method to rationally design RNA structures was proposed by [9]. The method, known as ‘RNA (archi)tectonics’ re-purposes fragments from natural RNAs to form prescribed 3D architectures. Its first success was a novel RNA ligase developed with the goal of understanding the structural constraints of ribozymes [10]. Rather than being fully rationally designed, however, it involved both rationally designed constant regions and variable regions which were evolved under *in vitro* selection.

Building on the success of these initial experiments, the first fully rationally designed RNA nanostructures were created by Jaeger, Westhof, and Leontis [11], which utilized the tertiary interaction of RNA molecules to form defined branched junctions which they called tectoRNAs. These early constructs were designed with integrated ligand and receptor loops which could bind to one another. Unlike biological RNA structures which self-assemble isothermally at 37°C, the tectoRNAs were assembled via an annealing protocol which involved temperatures as high as 90°C [11]. Paralleling the development of DNA nanotechnology in first forming individual defined junctions, then assembling those into defined periodic arrays, Chworos et al. [12] demonstrated the organization of individual RNA ‘tectosquares’ equipped with complementary tails into large molecular architectures. Severcan, Geary, Verzemnieks, Chworos, and Jaeger [13] further explored the design of the space of tectoRNA squares, using three different motifs which could be self-assembled under thermal annealing into structures similar to the 2004 paper with different sizes, thermal stability and yields. In 2010, the Jaeger group used an optimized version of their tectoRNAs to create cubic RNA scaffolds which could self-assemble isothermally, enabling the application of these designs under physiological conditions [14].

### Applications of tectoRNAs: pRNA and nanorings

Parallel to the development of methods for RNA nanostructure design and construction, applications of RNA nanotechnology also advanced, particularly through the contributions of the Guo group. Although their primary aim was not to develop RNA structures, while working on improving the efficiency of gene therapy delivery they developed sophisticated RNA nanostructures using the packaging RNA (pRNA) of the phi29 bacteriophage as their tectoRNA [15]. The pRNA monomers have two independent domains, a pair of hairpin loops which handle dimerization, and a long stem which contains the 5’ and 3’ ends. Based on extensive probing experiments and computational modelling [16], researchers in the Guo lab identified that the sequence of the stem-region is unimportant for assembly, opening the possibility to re-engineer these structures to deliver functional payloads. The size of pRNA trimers is in a therapeutic nanoparticle sweet spot of 10–50 nm: the structures are too large for fast renal clearance, have multivalent attachment to receptors on the target cell, yet are still small enough to be taken up through endocytosis [17]. These structures were able to isothermally assemble and demonstrated a high degree of stability at a range of temperatures and pH values [15; 18]. Their first application-targeted structures were based on the hand-in-hand interaction between up to three pRNA subunits and used to deliver therapeutic siRNA, which was attached to the 3’ end of each monomer, to cancer cells [19,20].

pRNA nanoparticles were later optimized through the design of complementary strands between three individual RNA oligomers to assemble them into defined three-way-junctions (3WJ) [21]. These junctions were used as building blocks to construct a variety of different 2D and 3D shapes including 2D rings and interconnected rings [22], 2D polygons [23], dendrimers [24], and polyhedra [25]. These were then functionalized with targeting and therapeutic moieties and their biodistribution, cell targeting and efficacies measured [26]. At present, pRNA-based designs and therapeutic concepts continue to be produced by the Guo lab and collaborators, however the design and application of these structures has essentially become a separate field from other RNA structure design paradigms, including RNA origami which is the primary focus of this review.

Another structure which has been used in siRNA therapeutic delivery are the nanorings developed by the Shapiro lab. In a series of letters, the group first proposed a kissing loop complex formed by *E. Coli* ColE1 regulatory RNAs – RNA I/II – could be used as a tectoRNA to form rings [27]. They then used MD simulations to verify the stability of the complex [28] and experimentally realized the design [29]. At this point, they began to focus on these structures as potential carriers for siRNA therapeutics. Taking both the ring structure from Grabow et al. [29] and the cubic structure from Afonin et al. [14], they functionalized the edges with siRNA extensions and demonstrated their efficacy *in vitro* [30]. They further optimized the ring structure for one-pot isothermal transcriptional assembly and demonstrated incorporation of 2’–fluorinated uracils by wild-type T7 RNA polymerase to improve nuclease resistance and circulation times [31]. In the previous letter, Afonin et. al. only showed knockdown of GFP fluorescence *in vitro*, they then followed up a few years later showing the diverse functional applications including gene knockdown, surface receptor targeting, external control through toehold-mediated strand displacement, and viral inhibition of these structures as well as simple *in vivo* knockdown experiments [32].

### Learning from DNA motifs

When compared with DNA nanotechnology techniques, RNA nanotechnology in the late 2000s was far behind in terms of structural size and complexity. By this point, the DNA community had already demonstrated complex patterns in tile assembly [33], as well as both 2D [34] and 3D [35,36] DNA origami with over 10,000 bases (for a detailed comparison between DNA and RNA origami, see section 2). The rapid development of DNA was facilitated by the relative ease of designing DNA motifs using only Watson–Crick–Franklin base pairs and double crossover (DX) junctions between helices. In response, the RNA nanotechnology field began to develop constructs whose structural motifs looked more like DNA, where canonical base pairing was almost exclusively used to define structures. One of the earliest works in this direction was done by Afonin, Cieply, and Leontis [37] who were the first to introduce paranemic crossover (PX) junctions, a classic DNA tile motif [38], to the world of RNA.

Later, the Mao lab developed more complex RNA tiling designs based on their previous work with 2D and 3D DNA tiles [39,40]. These could tetramerize through a complementary pairing of loop-to-tail and further dimerize via tail-to-tail interaction to finally form homo-octamers around three times larger than the 3D structures of Afonin et al. [14]. These structures, however required higher Mg^2+^ concentrations and multistep annealing [41].

This period also saw a number of DNA/RNA hybrid strategies implemented by various groups. Before creating fully RNA tiles in Yu et al. [41], the Mao group assembled RNA-DNA hybrid tiles with three-way and four-way junctions [42]. Both the Mao and Sugiyama groups created DNA/RNA hybrid origami where synthetic DNA staples were used to fold an *in vitro* transcribed RNA scaffold into the classic DNA origami structures of a 2D sheet and 6–helix bundle (6HB). These scaffolds were significantly shorter than the virophage-derived m13mp18 scaffold commonly used in DNA origami (7249 bases), having only 717 [43], or 1071 [44] RNA bases, but conclusively demonstrated that RNA could be folded using the same principles as DNA. More recently, Parsons et al. [45] demonstrated more complex DNA/RNA hybrid wireframe origami structures using up to a 1980 nucleotide RNA scaffold designed with the DAEDALUS [46] algorithm. The only implementation of all-RNA scaffold and staple origami structure, Endo, Takeuchi, Emura, Hidaka, and Sugiyama [47], demonstrated small origami structures – again a 2D sheet and 6HB – using RNA for both scaffold (720 bases) and staples. While these structures represent the first true ‘RNA origami’ (folding in a similar way as DNA origami with a scaffold and staple architecture), the laborious transcription and purification process for the many RNA staples from DNA templates meant that this technique did not achieve further adoption in the community.

### RNA origami

‘RNA origami’ as we know it today emerged from the Andersen lab in 2014 for which they also provided a new theoretical basis for the design and the *in vitro* transcription of RNA structures [48,49]. These RNA constructs were made up of ssRNAs with alternating stacks of kissing loop (KL) motifs and DX junctions arranging the strand into 2D corrugated sheets with sizes of a few hundred bases. These sheets could then assemble into larger finite-sized or lattice-based architectures. While these structures don’t follow the scaffold- and staple-construction method of DNA origami, the similarity in raster pattern and size makes the name appropriate, perhaps even more so since the single-stranded architecture is more akin to folding a single sheet of paper into a target shape without glue rather than ‘cheating’ by using staples. Impressively, these RNA origami were able to fold co-transcriptionally without a heating step, simplifying their production and opening the possibility of bioproduction at large scale [48].

While the Geary et al. [48] method of RNA origami design remains the most common, Han et al. [50] developed an alternate design paradigm for ssRNA origami. Building off the PX crossover motif of Afonin et al. [37], they demonstrated the creation of the largest RNA origami structures to date with over 6000 bases. These structures are essentially assembled as a single large hairpin with alternating paired and unpaired regions, the unpaired regions then fold back on themselves in PX crossovers, creating a single coherent sheet. Unlike the Geary et al. [48] method, however, the Han et al. [50] method involved thermal annealing from 85°C over 18 hours in a high-Mg_2+_ buffer; however, it was later demonstrated that similar structures could be annealed from 65°C in 10 hours without Mg^2+^ [51].

The current state of the art in RNA origami design and production is an updated version of the Geary et al. [48] method packaged as a software tool: RNA Origami Automated Design (ROAD) [52]. ROAD increases the size of designed origami up to the few-kilobase scale through providing a tool for analysing the potential barriers to folding and generation of optimized sequences (discussed further in section 3).

### Imaging structures with AFM

Atomic force microscopy (AFM) is a powerful and well known technique for observing individual structures with nanometre resolution. The imaging technique has provided unprecedented insights into the structure of biological macromolecules in their native environment and in real time with minimal sample alteration (labelling, fixing, or coating are not required) and has been applied to DNA structures since the first large-scale 2D arrays in the late 1990s [53] and to RNA since the early days of RNA tectonics [12]. High-resolution imaging can reveal fine details of the structures, such as the folding patterns [52,54] and the overall shape [55], which, if the tip radius is sharper than the feature under visualization, closely reflect the true profile. AFM has proven invaluable for DNA and RNA design as a method to verify correct assembly of structures, perform yield quantification, and measure properties such as persistence length. Although AFM imaging with its most advanced modes can offer a comprehensive 3D view of certain structures including cell membranes [56,57], in nucleic acid structures it is generally most applicable to flat or extremely stiff samples, as wireframe 3D DNA and RNA structures tend to collapse onto the charged imaging substrate due to electrostatic interactions and the intrinsic flexibility of nucleic acids [41].

### Imaging structures with cryo-EM

Cryogenic electron microscopy (cryo-EM) has recently become an invaluable validation tool in the molecular design field due to its ability to resolve structures in 3D. Advances in the technology have allowed resolutions of nucleic acid structures Angstrom resolution [58] and begin to understand the dynamics of designed DNA and RNA structures [59,60]. With cryo-EM revealing the molecular details of structures, the ability of molecular designers to correct defects and understand the real 3D geometries of their structures has been greatly improved. The combination of designed rigid scaffolds and functional moieties from nature has also allowed researchers to resolve new structural details of nucleic acids and RNA origami folds [61].

### RNA origami and cells

One of the main promises of the RNA origami field is the ability to amplify a relatively small amount of starting DNA template into significantly higher quantities of a specific RNA structure. For most of the history of the field, this was done using *in vitro* transcription with T7 polymerase. Another level of scalability can be achieved through bioproduction, where the DNA template can be amplified via cellular replication, and then the transcriptional machinery in cells again produces many RNA structures from each DNA template. Li et al. [62] successfully transcribed RNA structures from transfected DNA plasmids in *E. coli* that co-transcriptionally assembled into 2D and 3D tectoRNA structures up to 1500 bases in size which could then be purified from the cell lysate [62].

Alternatively, natively expressed RNA origami structures were used by Pothoulakis, Nguyen, and Andersen [63] to modulate eukaryotic cell behaviour. To accomplish this, they transformed *S. cerevisiae* with RNA origami expression cassettes which were transcribed by RNA polymerase III. These origami structures activated genetic circuits via an RNA origami complex which scaffolded an sgRNA+dCas9 targeting a specific genomic regions with aptamers which recruited transcription factors to the region specified by the sgRNA sequence.

**Figure 1.**
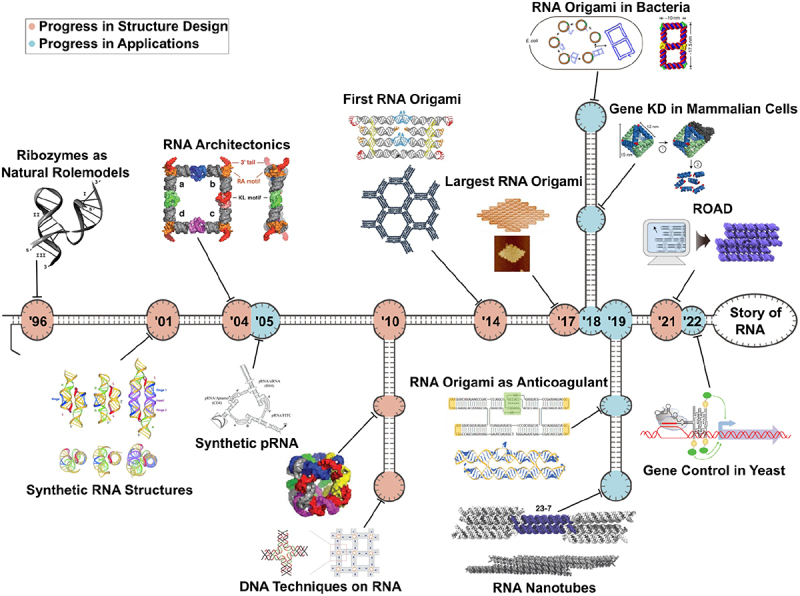
The history of RNA origami. With the first studies on the structural design of ribozymes in the late 1990s, researchers began to understand how to re–use natural structural motifs in synthetic RNA, with the basic constructs published in 2001 by Jaeger et al. [11]. Soon after, larger architectures were developed using “tectoRnas” – RNA with simple designs and interaction motifs based on natural RNA structures – to build arbitrary 2D and 3D shapes. In an early example of applications for synthetic RNA nanostructures, structures designed using pRNA were used to package siRNA therapeutics for antitumor therapy in mice. The complexity of RNA structures increased significantly with the application of fundamental principles from DNA nanotechnology, like multiway junctions and tile assembly, to create larger 3D structures. Nearly 20 years after the field’s beginnings, the first “RNA origami” co-transcriptionally folded from a single RNA molecule was developed by Geary et al. [48]. Following this major leap in its evolution, the field of RNA origami soon had larger structures, with the largest origami thus far created in 2017 by Han et al. [50]. Especially in recent years, great progress has been made in the bioproduction of RNA origami, with the first complex nanostructures produced in bacteria by Li et al. [62] and in eukaryotic cells by Pothoulakis et al. [63]. Not only production methods, but also general functionalization improved enormously, RNA origami were used as structural nanotubes and anticoagulants with clinical potential. Finally, with the introduction of ROAD by Geary et al. [52], the field was revolutionized through the easily applicable design workflow capable of generating kilobase structures which could fold co–transcriptionally. As the complexity of design increased, so to did the complexity of applications. Høiberg et al. [64] demonstrated delivery of multiple siRnas on a single structure and Pothoulakis et al. [63] demonstrated RNA origami which controlled gene circuits in *S. cerevisiae*. Figures adapted with permission from [9,11,12,28,48,50,52,62–66] Adapted from *RNA biology*, 1/4, Eric Westhof, Benoit Masquida, Luc Jaeger, RNA tectonics: towards RNA design, R78-R88, Copyright [9], with permission from Elsevier. **05** Adapted with permission from Khaled et al. [20]. Copyright 2019 American Chemical Society. **18a** Reprinted under a CC-BY license from M. Li et al. [62]. **18b** Adapted with permission. © 2018 WILEY-VCH Verlag GmbH & Co. KGaA, Weinheim. **19a** Adapted with permission. © 2019 WILEY-VCH Verlag GmbH & Co. KGaA, Weinheim. **19b** Adapted with permission from Stewart et al. [65]. Copyright 2019 American Chemical Society. **22** Adapted under a CC by license from Pothoulakis et al. [63].

Throughout the course of its development over the past few decades (Figure 1), RNA origami has demonstrated versatility in its design methods and applications. RNA origami offers some advantages over the more ubiquitous DNA origami, especially in terms of scalable and monitorable expression, co-transcriptional folding and more achievable isothermal assembly. Although its development faces multiple challenges, it is an exciting time in the field of RNA origami, with the introduction of improved design methods and complex biological applications, the field is set to experience a renaissance of structural innovation.

## RNA origami versus DNA origami

With these recent remarkable developments in RNA origami, some in the DNA origami community may ask whether it is time to switch. The history of RNA origami has taught us that RNA origami at its best does not fold just like DNA, and the choice of nucleic acid will certainly remain application dependent. We have collected a few aspects as key considerations to choose between DNA and RNA origami.

- **Achievable size**: The largest achieved RNA origami is the diamond structure from Han et al. [50] with 6320 nucleotides, which is slightly smaller than half the size of a DNA origami which uses the full m13mp18 scaffold (14498 nucleotides). For the foreseeable future, record-sized engineered nucleic acid nanostructures will still be made from one or multiple DNA origami. RNA structures still have a long way to go to catch up with the million-nucleotide finite and addressable structures of Wintersinger et al. [67].
- **Production**: Although progress has been made towards the biotechnological mass production of DNA origami [68,69] and expression in mammalian cells [70,71], the co-transcriptional folding of single-stranded RNA origami is inherently compatible with the existing genetic engineering toolbox. DNA templates for RNA origami can be commercially purchased as custom-synthesized gene fragments or plasmids for *in vitro* transcription or transcription in cells – making it an exciting tool for top-down as well as bottom-up synthetic biology.
- **Chemical functionalization**: Additional chemical functionalization is often required to impart function in DNA origami. The commercially available toolbox for chemical functionalization of synthetically manufactured DNA is continuously growing, and attaching e.g. cholesterol-tags for membrane anchoring or antigens for immunostimulation to DNA origami is often straightforward. When producing structures in cells, however, molecular engineers are limited to the biochemical toolbox of the cell. Here, the chemical versatility of RNA and its enzymatic modifications and protein interactions known from fundamental biology research are advantageous for adding function *in vivo* [72]. Therefore, DNA origami will, to a large extent, remain an externally added stimulant, while RNA origami is more likely to find its niche in structures produced and targeted to the intracellular environment by means of genetic engineering.
- **Annealing**: Due to the kinetics of multi-stranded assembly, DNA origami is typically annealed by heating to 70°C-90°C followed by a cooling process over the period of hours. Recent progress has been made towards isothermal assembly of DNA origami, but folding remains slow [73]. On the other hand, single-stranded RNA origami structures which take inspiration from natural RNA structures’ ability to co-transcriptionally fold can assemble in high yield at 37°C within minutes.
- **Computational tools**: Computational design tools for RNA origami are becoming increasingly available and usable, though they still lag behind the variety and sophistication of the tools available for DNA origami. Due to the biological relevance of solving the RNA folding problem, RNA structure prediction algorithms are more numerous and developed than DNA; however, the molecular dynamics simulation of RNA origami structures is still in its infancy (see section 4).
- **Visualization**: All applications of RNA and DNA origami – present and future – benefit from straight-forward strategies to incorporate fluorescence visualization. While DNA origami has to be labelled with fluorophores during staple synthesis, it is trivial to extend loops or strand ends of RNA structures with fluorescent RNA aptamers, enabling visualization without covalent tagging. Recent developments include fluorescent RNA aptamers compatible superresolution microscopy [74].
- **Catalytic activity**: While DNA can have rudimentary catalytic capabilities, dynamic DNA nanostructures are typically operating in or driven towards thermodynamic equilibrium. The straightforward integration of RNAzymes provides more opportunities towards the construction of chemically active molecular machines from RNA origami.
- **Stability**: If a stable molecular canvas is desired, DNA origami will remain the method of choice. However, early biomedical applications demonstrate that compact RNA origami is comparatively stable, e.g. compared to mRNA, and stability has not been reported as a major issue [51,75].

## Computational design tools for RNA origami

Compared to DNA origami, where structures generally fold as designed after annealing and a diverse array of computational design tools are available, designing RNA architectures is challenging due to the complexity and variety of RNA motifs and relatively few computer-aided design tools. At the same time, it is clear that computational tools for design are key drivers to accelerate the growth of the RNA origami community. Therefore, we will review the status quo and key advances concerning computational tools for RNA origami design and structural prediction.

### Fragment assembly and optimization

**Figure 2.**
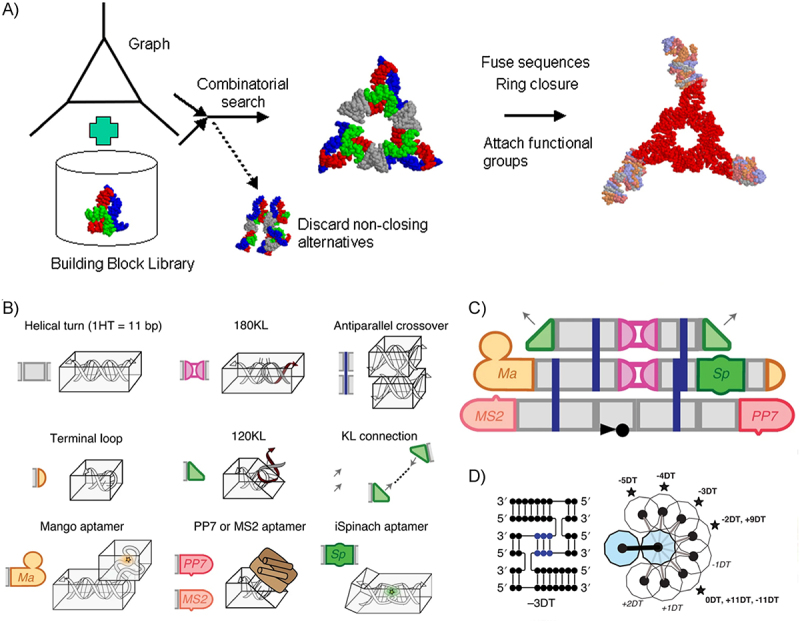
RNA origami design. A) Early software optimization of RNA ring structures was performed with tools like Nanotiler [76]). B-D) the ROAD software enables the design of co–transcriptional RNA origami. B) ROAD implements several structural RNA motifs. C) the combination of these motifs allows for the design of complex architectures. D) in particular, dovetail seams, in which crossovers are offset by numbers of bases differing from a full A-helical turn enable the generation of 3D structures. A) Reprinted from Journal of Molecular Graphics and Modelling, Vol 27, E. Bindewald et al., Computational strategies for the automated design of RNA nanoscale structures from building blocks using NanoTiler, Pages 299–308, Copyright (2008), with permission from Elsevier. B-D) Figures adapted with permission from [52].

Early attempts at software optimization of RNA nanostructures focused on tools like Nanotiler, which used a computational pipeline to generate RNA building blocks, with a particular focus on optimizing RNA ring structures (Figure 2A [76],). Recently, Yesselman et al. developed the RNAMake software, which offers an automated tool to improve the stability of tetraloops and optimize RNA aptamer structures, based on 3D RNA structures available in a public database [77].

### Early RNA origami design methods

The first design pipeline for single-stranded RNA origami which co-transcriptionally fold was developed by Geary et al. [48]. Rather than a single tool, Geary et al. [48] used Assemble2 [78] to build up structures based on double-crossover (DX) motifs with various geometries [49]. The structures were then sequence-optimized for co-transcriptional folding using hand-edited text schematics, custom Perl scripts and NUPACK [66].

When designing the large PX RNA origami structures, Han et al. [50], used the free-form DNA and RNA design software Tiamat [79]. Free-form design allows much more flexibility in geometries, however can be extremely tedious due to the need to define every component. Designing large structures in Tiamat works best when there is a simple motif (the alternating hairpin and PX regions in this case) which can by copy-pasted throughout the design. Tiamat also has robust sequence assignment tools to avoid repeated sequences in the structure. Li et al. [62] also used Tiamat to design their co-transcriptionally folded RNA wireframe structures. In addition to Tiamat, they iteratively used the thermodynamic folding model mFold [80], to optimize the sequence to avoid spurious interactions.

### Co-transcriptional RNA origami with ROAD

A significant improvement in RNA origami design was the release of the open source software RNA Origami Automated Design (ROAD), developed by Geary et al. [52] to design co-transcriptional RNA origami in a more robust and streamlined fashion. ROAD formalizes the creation of text-based schematics of RNA origami made up of double-crossover motifs and kissing loops which form 180° and 120° angles between RNA helices. It also supports the functionalization of designed structures with various aptamers (Figure 2B). Using these building blocks, Geary et al. [52] synthesized a variety of 2D RNA origami structures that self-assemble into larger nanostructures (Figure 2C).

The structures designed by ROAD are quite similar to the single-layer ‘lattice-based’ designs created with Cadnano [36] with many parallel helices connected by double-crossover junctions. These junctions define the key structural motif of ROAD-designed RNA origami: the ‘dovetail seam’ – the number of base pairs between crossovers to neighbouring helices from an individual helix. The spacing in the dovetail seam determines the angle in 3D space and the vertical alignment between connected helices, formalizing the design of flat or curved structures (Figure 2D [60]). Due to the periodicity of the RNA A-form helix, dovetail seams can only define 11 discrete interhelical angles based on the integer number of base steps between crossovers. To ensure co-transcriptional folding, ROAD uses Revolvr, an iterative sequence optimization algorithm: First, the ViennaRNA [81] package is used to calculate the minimum free energy structure (MFE) of a candidate sequence. If the minimum energy is not achieved, the sequence is then mutated and the procedure is iterated until the desired structure is found to be the minimum free energy structure. The key optimization performed is improving the orthogonality of paired sequences, preventing spurious base-pairing which would result in an incorrect structure. This automated approach is similar to the manual approach using mFold used in Li et al. [62]. The RNApath program included in the package annotates regions of the structure where the strand routing would cause duplexes to form after nearby hairpins, representing potential kinetic traps which may impede assembly. The Andersen lab has demonstrated a large variety of structures designed using ROAD [52,60,82], highlighting the robustness of the algorithm and paving the way for a new generation of RNA origami design. Although the developments are promising, the engineering of 3D RNA architectures is still challenging – ROAD does not facilitate the creation of more complex architectures such as multi-armed junctions, layered, or intrinsically curved structures. As the field matures, we expect to see further developments in RNA origami design software, and closer coupling to physics-based computational methods, which will be the focus of the next subsection.

### Wireframe RNA design methods

Another method inherited from DNA origami design, algorithmic routing of strands on polyhedral meshes has recently been used to design single-stranded [83] and DNA/RNA hybrid origami [45]. In both cases, the input to the algorithms is a 3D polyhedral mesh generated from 3D rendering software (Blender in the case of Elonen et al. [83], anything that can generate the common .ply file type in the case of Parsons et al. [45]). The routing algorithms, Sterna [83] and pyDAEDALUS [45] then decompose the 3D mesh into vertices and edges and computes the spanning tree of the mesh. In the case of Sterna, each edge will end up a single RNA helix, which makes certain polyhedra impossible to fully route as a spanning tree. These edges are filled in later using the same kissing loop motifs used in ROAD designs. PyDAEDALUS is an extension of the older DAEDALUS algorithm [46] which only worked for DNA, and uses two helices per edge which fulfils Euler’s theorem for connected graphs such that the scaffold can be routed to cover each edge twice. To facilitate single-stranded folding, Sterna uses ‘geodetic ordering’ where each vertex is evaluated to ensure that strands always enter and leave the vertex through neighbouring edges to avoid tangles. These routing methods have proved to be very robust and scalable for generating wireframe DNA origami structures with arbitrary geometries, time will tell if these initial demonstrations of the method trigger a new generation of wireframe RNA structure design the way ROAD has for lattice-based designs.

## Computational modelling tools for RNA origami

Computational tools are often employed in nucleic acid nanotechnology contexts to obtain physics-based estimates of stability and mechanical properties of the designed structures. While not as common as their DNA counterparts, there have nonetheless been some studies of designed RNA nanostructures using computational methods. Broadly, there are two common methods used to study RNA structures: thermodynamic/kinetic folding models, and molecular dynamics (MD) simulations. Folding models are useful for predicting if a given sequence is probable to fold into the desired 2D or 3D shape, while MD simulations are used for studying the mechanical properties of structures, generally assuming correct folding.

### Thermodynamic and kinetic folding models

Folding models attempt to predict the folding pathway and/or final 2D or 3D structure of an RNA sequence using physical first-principles of RNA base interactions. These models are not unique to designed RNA nanostructures – most were built with the intention of predicting the structures of natural RNAs. However, since the fundamental base-pairing interactions are the same, the models transfer well between the two domains (and in fact often work better for designed structures where there are fewer tertiary interactions). The most popular models used today are all dynamic programming approaches based on the algorithm developed by Zuker and Stiegler [84] which use the Turner model [85] of thermodynamic parameters for dinucleotide steps [80,86,87]. This model uses the data from extensive optical melting experiments to define the thermodynamic stability of each two-nucleotide step along the helix as well as the entropy-associated stability loss from closing a loop. For a comprehensive introduction to nearest-neighbour models, see Andronescu, Condon, Turner, and Mathews [88].

The dynamic programming implementations of the nearest-neighbour model, while fast and relatively accurate, have the fundamental limitation that they can only predict non-pseudoknotted structures (that is, all base pairs are nested). This is because pseudoknots violate the dynamic programming assumption that substructures in previous layers of the recursion are already optimal [89]. Furthermore, enumeration of potential pseudoknotted states is an NP-complete problem [90], and therefore not computationally tractable beyond very short RNA structures. Some algorithms have attempted to use heuristic [91] or global pairing probabilities [92] approaches to make pseudoknot-prediction possible, however these algorithms have not seen widespread adoption in the RNA nanotechnology community. Recently, machine-learning models trained on databases of known structures have begun to outperform purely physical models for natural RNA structure prediction [93,94]. These have not been applied in the context of RNA nanotechnology, largely because it is expected that their accuracy will be low since designed structures look quite different from the natural RNAs these models were trained on.

In practice, the simple dynamic programming-based thermodynamic models are used for sequence design to minimize crosstalk between sections of designed RNA structures and optimize loop energy to control assembly kinetics. This has been a part of RNA nanotechnology from the very beginning; Jaeger et al. [11] used the mFold implementation of the Zuker and Stiegler [84] algorithm to optimize their TectoRNA sequences. Recent RNA design algorithms still use the successors to these models during the design process. For example, Revolvr in the ROAD package uses RNAfold [81,84], while Sterna uses NUPACK [86] to verify that each non-pseudoknotted component of the overall design is likely to fold into the target structure.

### Molecular dynamics (MD) simulation

MD simulations are often used in the nucleic acid nanotechnology field to understand the dynamic mechanical properties of designed nanostructures. Because of the nanoscopic size of these structures, direct visualization of individual particles at nucleotide resolution is currently impossible, with the latest developments in individual-particle cryo-electron tomography (IPET) on RNA origami reaching almost 20 Å [95], while obtaining high-resolution structures via cryo-EM requires ensemble averaging of hundreds of thousands of individual particles giving local resolutions as high as 3.4 Å [60]. MD simulations provide computational support through which the behaviour of an individual nanostructure can be investigated in an approximate fashion.

**Figure 3.**
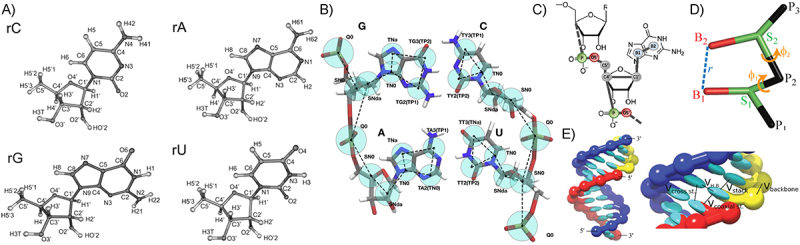
Computational modelling of RNA origami with different levels of coarse-graining. A) All-atom force fields specify interaction potentials between each atom in the RNA molecule. B) the general-purpose MARTINI force field coarse-grains each RNA nucleotide into six pseudoatoms. C) HiRE-RNA uses either six or seven coarse-grained beads per nucleotide. D) TIS (three interaction site) places beads at the centers of mass of the sugar, the phosphate and the base site. E) oxRNA uses a single anisotropic bead per nucleotide with empirically–derived interaction potentials between the beads. A) Adapted with permission from Zgarbová et al. [96] Copyright 2013 American Chemical Society. Further reprint permission inquiries should be directed to ACS. B) Reprinted from *Biophysical Journal*, 113/2, Jaakko J. Uusitalo, Helgi I. Ingólfsson, Siewert J. Marrink, Ignacio Faustino, Martini Coarse-Grained Force Field: Extension to RNA, 246–256, Copyright [97], with permission from Elsevier. C) Adapted with permission from Pasquali and Derreumaux [98] Copyright 2010 American Chemical Society. D) Adapted with permission from Denesyuk and Thirumalai [99] Copyright 2013 American Chemical Society. E) Reprinted from Šulc et al. [100], with the permission of AIP publishing.

Readers are likely most familiar with all-atom MD simulations, where every atom in the structure is explicitly represented (Figure 3A). The average RNA origami, however, is thousands of bases in size, which is too large to be simulated in a reasonable timeframe by all but the most powerful computers. Additionally, when researchers do run all-atom simulations on RNA structures, RNA has proven to be a difficult molecule for all-atom force fields to represent and requires multiple additional fixes on top of the base force field to achieve the correct A-form helix geometry [96,101].

To overcome the challenges associated with all-atom modelling, numerous coarse-grained models of RNA have been developed which work at a variety of levels of resolution (Figure 3C-E) [98–100,102–106]. Of these, however, only oxRNA (Figure 3E) [100] has achieved limited adoption in the nucleic acid nanotechnology community due to its efficient GPU implementation [107,108] and relative ease of installation and use. oxRNA has been used to study a few designed RNA structures [83,109,110], primarily with the goal of understanding how design choices influence the resultant structures. There has also been one paper [111] which used simRNA, a coarse-grained force field for Monte-Carlo sampling of RNA structure [102,112], to optimize kissing loop placement and orientation. The other coarse-grained RNA models have, thus far, only been applied to natural RNA structures. It should be noted that the general-purpose coarse-grained model, MARTINI, supports RNA (Figure 3B) [97]; however, its accuracy remains below that of the RNA-specific models and improving it is an ongoing area of research. For a comprehensive overview of RNA simulation methods, see Sponer et al. [113]. All in all, advances in the simulation tools for RNA origami are expected to inform and refine its design and should thus be developed alongside experimental approaches.

## Functional RNA origami

Alongside the development of the structural motifs and computational design tools, the use of RNA origami in biomedical applications has also begun to mature. RNA origami provides new possibilities in nanoscale positioning as well as new biophysical tools to control and manipulate cells. In this section, we review recent remarkable examples that give a flavour of future possibilities.

### Biomimetic molecular hardware

**Figure 4.**
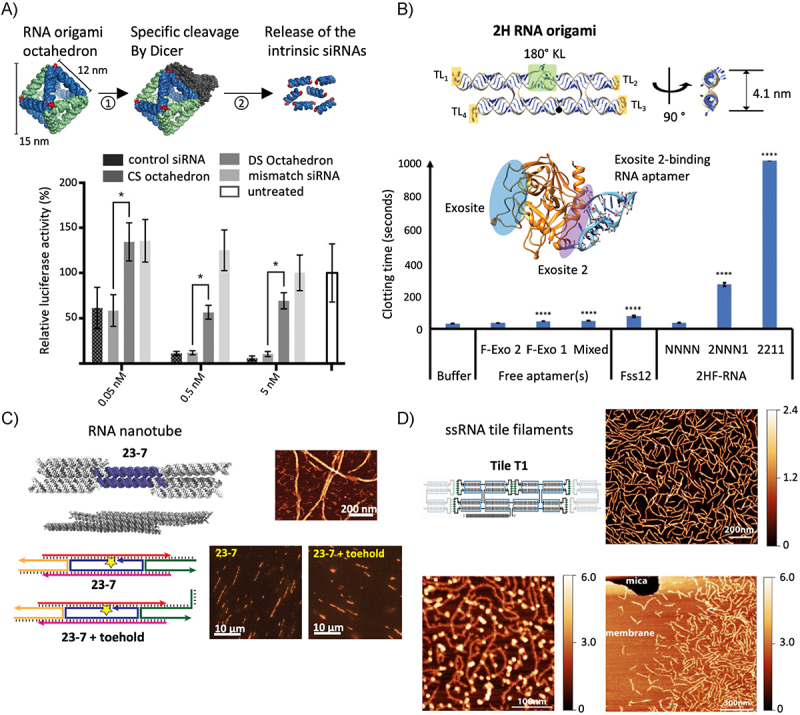
Molecular hardware. A) Schematics of an RNA octahedron design (top) and gene knockdown experiment (bottom) showing the high activity of the RNA octahedron in a dual luciferase assay in H1299 cells [64]. B) 3D model of a 2–helix RNA origami (2HF–RNA) with thrombin aptamer binding sites highlighted in yellow (top); schematic from the crystal structure of an exosite 2-binding RNA aptamer bound to thrombin and PTTe assay showing the increased anticoagulant activity upon action of 2HF-RNA displaying four aptamers (2HF-RNA2211) (bottom) [115]. C) Design (left), AFM (top right) and epifluorescence microscopy characterization (bottom right) of micrometer-sized nanotubes without and with toehold [65]. D) design and characterization of RNA filaments showing AFM images of filaments (top right), and its binding either to proteins (bottom left) and, in presence of biotin aptamers, to biotinylated lipid membranes (bottom right) [116] A) Adapted with permission. © 2018 WILEY-VCH Verlag GmbH & Co. KGaA, Weinheim. B) Adapted with permission. © 2021 Wiley-VCH GmbH. C) Adapted with permission from Stewart et al. [65]. Copyright 2019 American Chemical Society.

RNA nanostructures have been used to construct molecular components which mimic proteins in form and function. Inspired by the DNA origami nanopores, cholesterol anchors were bound to 6–stranded RNA nanotubes to enable insertion into lipid membranes. These RNA nanopores could then be used to detect positively charged peptides [114]. Additionally, Stewart et al. [65] rationally designed multi-stranded RNA tiles to create biomimetic cytoskeletal nanotubes *in vitro* (Figure 4C). In addition to forming multimicrometre filaments, these structures could also be designed with overhangs, which would enable funcationalization or dynamic (dis)assembly via toehold-mediated strand displacement. De Franceschi, Hoogenberg, and Dekker [116] followed a similar strategy, this time with ssRNA origami tiles to assemble filaments hundreds of nanometres in length with tunable properties. To achieve mimics of cytoskeletal properties, they introduced membrane binding through functionalization with biotin aptamers which could bind to biotinylated lipids. To mimic the capping and disassembly of cytoskeleton, they designed additional ‘capping tiles’ lacking outgoing kissing loops and introduced a system where filaments could be dissassembled through strand displacement reactions which block loop–loop interactions. Finally, binding sites were added to recruit proteins which regulate the polymerization of cytoskeletal filaments (Figure 4D).

### RNA nanostructure bioproduction

**Figure 5.**
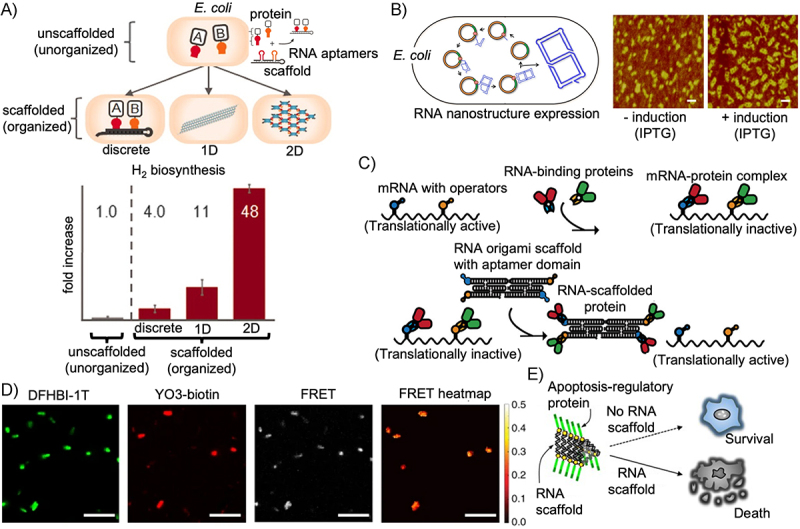
Expression and function of RNA origami in cells; A) (top) Synthetic RNA molecules assemble into functional discrete, 1D, and 2D scaffolds *in vivo* to control the spatial organization of ferredoxin and hydrogenase enzymes for hydrogen production. Single RNA molecules were folded into a duplex with aptamer loops which bound with PP7 and MS2 adaptor domains on the enzymes. The proteins and RNA scaffold were co-expressed in *E. coli* and the biosynthesis of hydrogen gas was monitored using gas chromatography. (Bottom) Hydrogen biosynthesis as a function of RNA scaffold organization. Hydrogen production increased up to 48–fold when the cells expressed the 2D scaffold [118]. B) Cloning and expression of RNA nanostructures *in vivo*. An expression vector (orange) carrying a DNA sequence (green) is transformed into *E. coli*. Upon IPTG induction, the DNA was transcribed into RNAs, which self–assemble into the designed double-square structure inside the cell [62]. C) RNA scaffolds were designed with several aptamers for the produced protein, which acted as a negative feedback self–regulating system. Expression could be recovered by expression of an RNA scaffold with competing aptamer domains [119]. D) Apta–FRET constructs were genetically encoded and transformed *E. coli* cells. When the RNA origami folded correctly, two fluorescent RNA aptamers were organized in close proximity, as a result, high FRET observed between the two fluorophores, DFHBI-1T and YO3-biotin [120]. E) Synthetic RNA scaffolds expressed in mammalian cells used to control cell-death pathways by regulating the assembly and oligomerization of apoptosis-regulatory proteins [121]. A) from Delebecque et al. [118]. Reprinted with permission from AAAS. B Reprinted under a CC-BY-NC-ND licence from Nguyen et al. [119]. C) Reprinted under a CC-BY license from M. Li et al. [62] D) Reprinted under a CC-BY license from Jepsen et al. [120] E) Reprinted under a CC-BY license from Shibata et al. [121].

One of the major advantages of RNA origami over DNA origami is the ability to produce the structures inside living cells in a straight-forward manner. For example, Li et al. [62] and Liu et al. [117] demonstrated transcription and folding of simple single-stranded 2D and 3D RNA shapes in *E. coli* after transformation with the corresponding DNA plasmid (Figure 5B). In another example by Jepsen et al. [120], fluorescent RNA origami structures demonstrating FRET-mediated energy transfer were also expressed in *E. coli* cells (Figure 5d). RNA origami have, at this point, not been produced in mammalian cells; however, Litke and Jaffrey [122] demonstrated expression of a designed, multivalent aptamer structure in mammalian cells using the twister ribozyme to achieve a high yield of circularized aptamer structures which were more stable than their linear counterparts [122]. The ability to produce these structures in bioreactors presents a relatively direct path towards scaling up expression of functional RNA origami structures for applications in biotechnological and biomedical settings.

### RNA nanostructure biodistribution

Another largely open question, particularly in biomedical applications, is the biodistribution and pharmacokinetics of systemically delivered RNA origami therapeutics. Short-circulating particles are sometimes preferred to decrease toxicity, while in other cases, long-circulating particles are beneficial for slow-acting therapies [17]. RNA is an excellent biomaterial for nanoparticle construction due to its tunable size, which is a major determinant of circulation time. In a study by Jasinski et al. [75], the circulation time of an RNA nanoparticle was shown to increase as the size changed from 5 to 25 nm in whole-body images of tumour-bearing nude mice over a 24–hour period.

The ability to distribute RNA nanoparticles systemically has important implications for the development of RNA therapeutics. For example, an RNA origami anticoagulant developed by Krissanaprasit et al. [123] combines the controlled multivalency and nanoscale positioning which RNA origami enables. The RNA origami nanostructure contains multiple thrombin-binding aptamer sequences, which due to the scaffolding showed increased anticoagulant activity in activated partial thromboplastin time (aPTT) assays compared to thrombin-binding aptamers alone. Furthermore, these anticoagulants could be rapidly inactivated by systemic application of DNA strands which disrupted the structure of the aptamers via toehold-mediated strand displacement (Figure 4B) [115].

### Metabolic engineering

Much of the regulation that occurs in cells requires the recruitment of many independent molecules to sites of transcription and translation. Furthermore, many reactions in cells require multiple independent enzyme-catalysed reactions, which can be accelerated through localization of the enzymes. One of the most promising *in vivo* uses of DNA and RNA structures is to scaffold other active molecules to improve control and increase efficiency of metabolic processes through ‘substrate channelling’. As an example of substrate channelling, RNA nanostructures were expressed in *E. coli* to assemble into discrete, 1D, and 2D scaffolds functionalized with protein-docking sites, which were used to control the spatial organization of proteins involved in the hydrogen biosynthesis pathway *in vivo* (Figure 5A). This scaffolding resulted in a 48–fold increase in hydrogen production [118].

In a study by Nguyen, Pothoulakis, and Andersen [119] the researchers designed mRNAs with internal operator sites which repressed translation upon binding of the protein product (Figure 5C). The expression of the protein could then be increased by co-expression of an RNA origami scaffold which contained competing aptamers which sequestered the product. This approach was used to control the deoxyviolacein metabolic pathway by balancing the enzyme expression using an RNA regulator, which is an attractive tool for RNA-based regulation of gene expression in synthetic biology.

### Controlling gene expression

In an early example of controlling cell behaviour, small, synthetic RNA scaffolds were used to control apoptosis by detecting and assembling an apoptosis regulatory protein, caspase-8 (Casp-8). By using the ability of RNA nanostructures to position proteins with nanometre precision, apoptosis could be induced via transfection of the RNA scaffolds (Figure 5E) [121].

More recently, a functional octahedral RNA origami was designed with intrinsic substrates for Dicer in order to produce multiple siRNAs that can be released with precise stoichiometric control [64]. The origami caused efficient silencing of the Renilla luciferase target gene as was observed via dual luciferase assay of H1299 cells transfected with the origami (Figure 4A).

A more complex example of scaffolding for cell behaviour control took inspiration from Cas9–mediated gene activation/inactivation. Catalytically inactive Cas9 (dCas9) is a popular tool for targeted gene modulated through the single-guide RNA (sgRNA)-directed, site specific recruitment of activating or repressing protein domains in yeast and mammalian cells. SgRNAs were fused with an RNA origami structure (termed sgRNAOs) containing multiple functional motifs which controlled the expression of genetic targets in *S. cerevisiae* yeast [63].

### RNA origami as an immune system stimulant

Several RNA nanoarchitectures have been described to function as immune stimulants and therefore a promising platform for immunotherapy applications. In a study by Qi et al. [51], single-stranded RNA origami were annealed in a physiological buffer, phosphate-buffered saline (PBS), without the addition of Mg^2+^ making them suitable for *in vivo* applications. The origami were then injected intraperitoneally into mouse models of peritoneal metastasis and peritoneal carcinomatosis. The RNA origami halted tumour growth or even caused tumour regression by stimulating the innate immune response through the Toll-like receptor 3 (TLR3) pathway. They were less toxic compared to polyinosinic/polycytidylic acid (PolyIC). RNA origami induced long-lasting antitumor immunity in presence of CD8 T and NK cells, which could potentially be explored as an effective and safe immunotherapeutic for treating cancer.

In another study by the same group on RNA origami as a TLR3 agonist, RNA origami decorated with antigens were used as a hybrid antigen/adjuvant for initiation of T-cell mediated immunity. These RNA origami-based vaccines were reported to induce anti-tumour immune response that contributed to long-term tumour immunity in the B16-OVA (ovalbumin) murine melanoma tumour model [124].

## Conclusion

RNA origami has made considerable progress towards a technology which may soon be as widespread as DNA origami already is. The use of RNA has several advantages compared to DNA origami due to its greater structural and functional versatility, for example concerning the integration of catalytic activity. This makes it an exciting tool for bottom-up as well as top-down synthetic biology. As we progress further in the production of functional RNA origami, we may see a rise in RNA origami-based hardware for synthetic cells. Simultaneously, it will be exciting to explore the therapeutic potential, which goes far beyond current RNA therapeutics thanks to the possibilities that come with structural versatility and spatial organization of secondary components.

There are many challenges that the RNA nanotechnology field must overcome to become a mature part of the biochemical toolbox. These can be broadly broken down into three categories:

1. The need for a more robust suite of design tools which offer friendly UI/UX experiences, enabling non-experts to leverage the power of designed RNA structures for their areas of interest rather than relying on domain experts who know the quirks and limitations of their favourite piece of software. Furthermore, better kinetic folding and MD simulation methods which are able to accurately model the complex, (pseudo)knotted folding pathways of RNA nanostructures are necessary to avoid costly and frustrating experimental re-engineering.
2. A deeper understanding of how RNA structures interact with living systems, especially the immune system. There has been some progress in this direction [51,125], laying out some basic rules for engineering innate and adaptive immunogenicity. However, before these structures can become widely used in biological systems, a more comprehensive understanding of off-target effects and pharmacokinetics are required.
3. Improved production and purification methods. In order for RNA structures to be viable, especially as therapeutics, researchers need to achieve faster, cheaper, and higher fidelity production of structures. Based on the breakthroughs in DNA synthesis over the past 20 years [126,127], there is reason to expect that RNA production, both synthetic and biological, will see a similar trend in the coming years.

Looking at the momentum that the RNA origami field is gaining, it is interesting to ask: Will RNA origami replace DNA origami? Or will both technologies coexist? We believe that both technologies will continue to grow and find new applications in their respective niches. DNA origami will likely remain superior as a stable molecular breadboard to position secondary components. RNA origami will conquer the field of synthetic biology as the method of choice to build functional hardware co-transcriptionally. We are also likely to see additional approaches similar to that demonstrated in Parsons et al. [45] where long RNA strands are folded using DNA staples to merge the advantages of functional RNA with the cheap synthesis and chemical modification of short DNA strands. Both are equally interesting for diagnostic and therapeutic applications and we may be curious to witness how origami made from DNA and RNA matures from nanoscale art to a transformative technology.
